# Synthesis of Glycerin Carbonate by an Environmentally Friendly Novel Catalytic Membrane of Poly(vinyl alcohol)/Deep Eutectic Solvent

**DOI:** 10.1021/acsomega.5c10125

**Published:** 2026-03-04

**Authors:** Guler Hasirci, Nilufer Durmaz Hilmioglu

**Affiliations:** 52980 Chemical Engineering Department of Kocaeli University, Izmit 41001, Kocaeli, Turkey

## Abstract

A novel and eco-friendly catalytic material, poly­(vinyl alcohol)-supported deep eutectic solvent (PVA/DES) catalytic membrane, was prepared and used to produce value-added glycerin carbonate (GLC) from glycerin and dimethyl carbonate (DMC) for the first time. The heterogeneous form of catalytic membrane was prepared in the form of a film using PVA polymer as a support and an environmentally friendly DES, consisting of potassium hydroxide (KOH) and poly­(ethylene glycol) (PEG), as the catalyst. Fourier-transform infrared (FTIR) and thermogravimetric analysis (TGA) characterizations were performed to assess the functional groups and thermal stability of the PVA/DES catalytic membrane. Moreover, the change in the chemical structure of the catalytic membrane after the reaction was investigated by FTIR. The interaction between the catalytic membrane and glycerin is described by contact angle measurements. The relationship between reaction time, amount of catalyst, temperature, DMC/glycerin initial molar ratio, and reaction yield in batch reactions with PVA/DES catalytic membrane was discussed. The maximum GLC yield of 65.4% was obtained at 33% catalyst amount, 85 °C, DMC/glycerin: 3:1, and a reaction time of 4 h. The additional membrane reactor study increased the 3 h GLC yield to 74.0%. Furthermore, the effects of catalyst amount and DMC/Glycerin ratio on the reaction yield in the Response Surface Methodology (RSM) study with a central composite design were compared. It was found that the PVA/DES catalytic membrane is a suitable and green catalytic material for GLC synthesis from DMC.

## Graphical abstract

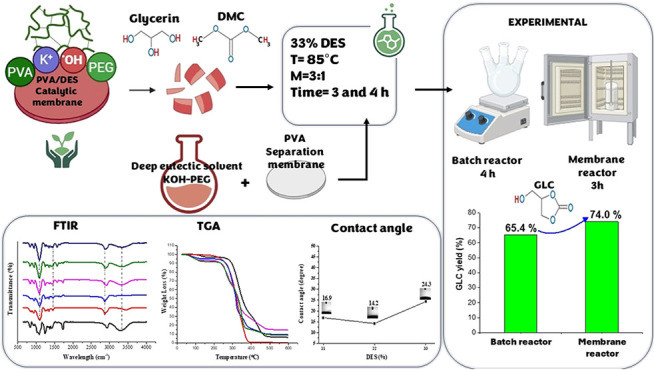

## 1 Introduction

Since the Industrial Revolution, the negative effects of fossil fuel use on the environment have reached alarming levels.^1^ Therefore, there has been an increase in the use of renewable fuels such as biodiesel as an alternative to fossil fuels for safer and more eco-friendly energy use.^2^ Increasing biodiesel production worldwide has increased the amount of crude glycerin produced as a byproduct, reducing its unit selling price. Glycerin, a versatile molecule, can be transformed into many value-added products through various reactions. GLC can be produced under environmentally friendly conditions by transesterification from glycerin and DMC. It attracts attention with its potential to create added value.^3,4^ Principally, GLC (4-hydroxymethyl-2-oxo-1,3-dioxolane) is a versatile, low-toxicity, and biodegradable molecule with reactivity versatility.^5^ It has a wide range of applications in industries such as green solvents, dyes, chemicals, and biopolymers.^6^ Furthermore, it can be used as an environmentally friendly fuel additive and an electrolyte in lithium-ion batteries for cleaner and safer energy use.^7,8^ For the GLC synthesis from DMC, heterogeneous catalysts are preferable to homogeneous catalysts due to their easier recovery from the reaction and reusability.^9^ Lian et al. used mixed oxide catalysts (CuO/ZnO/MnO_2_) produced by the coprecipitation method for DMC-glycerin transesterification. MnO_2_ in the catalyst acted as a support, increasing the adsorption capacity and the concentration of basic sites. They achieved a maximum conversion of 99.7% glycerin at 90 °C for 90 min, with a DMC/GL ratio of 5 and 3% catalyst loading.^10^ Sert and Sert used the heterogeneous catalyst obtained by adding calcined waste mussel shells to the biocoal, prepared from the pyrolysis of used coffee grounds at certain temperatures (400, 500, 600 °C), in the GLC synthesis from glycerin and DMC. They observed the highest catalytic performance in the catalyst prepared at a pyrolysis temperature of 600 °C. The calcined waste mussel shells contributed to the catalytic activity by increasing the number of basic sites in the catalyst. They obtained a maximum conversion of 58.5% at 75 °C, M: 4/1, and 4% catalyst loading.^11^ Catalytic membranes produced by combining membrane systems and catalysts are attractive candidates as heterogeneous catalysts for GLC synthesis.^12,13^ During the preparation of the catalytic membrane, the catalyst can be embedded in the membrane or coated on its surface.^14^ Polymers are often used as matrices in the production of catalytic membranes and can be functionalized with catalysts, serving as heterogeneous catalysts in the reaction process.^13^ PVA is a versatile polymer used in catalytic membrane preparation due to its good film-forming properties, high hydrophilicity, low cost, and biodegradability.^15^ PEG, another green and eco-friendly polymer, is a versatile, highly hydrophilic substance widely used in the food industry, biomedical industries, and pharmaceutical applications. Functionalized PEG can play an active role in reaction catalysis by acting as a proton acceptor or donor.^16,17^ It is also an environmentally friendly hydrogen bond donor in DES preparation. DESs are mixtures formed by the hydrogen bonding interaction of their components and have a lower boiling point than the individual components.^18^ In two-component DESs, one component is a hydrogen bond acceptor, and the other is a hydrogen bond donor.^19^ DESs are cheap, safe, biocompatible, highly biodegradable, low-volatile, environmentally friendly, property-tunable, and a green alternative to toxic organic solvents.^20^ DESs can serve as catalysts in reactions such as esterification, organic synthesis, glycolysis, and depolymerization. When used as catalysts, they have significant advantages such as ease of recovery, nontoxicity, catalytic effects similar to those of acids, and reusability.^21^ Wang et al. used a DES catalyst produced from KOH and monoethanolamine (MEA) at different molar ratios (1:2, 1:3, 1:4) in the glycidol and GLC synthesis from glycerin and DMC. A maximum GLC yield of 29.4% was obtained under a 1:4 KOH/MEA molar ratio, 80 °C reaction temperature, 3% catalyst amount, and 3:1 DMC/glycerin molar ratio.^18^ Very few studies use polymeric membrane structures as heterogeneous catalysts for synthesizing GLC from DMC. Hasirci and Hilmioglu synthesized GLC from DMC with a heterogeneous catalytic membrane obtained by adding sodium methoxide as a catalyst to the PVA/PVP blend as a polymeric matrix. The effect of the reaction parameters, such as temperature, reaction time, DMC/glycerin molar ratio, and amount of catalyst, on the reaction yield was investigated. They achieved a 99.7% GLC yield at 75 °C with 12.5% catalyst loading and DMC/glycerin = 3:1 for 3 h.^22^ Although the use of DES as a catalyst in GLC synthesis from DMC is relatively new and limited, DESs have been used in a homogeneous form in this reaction.^18^ In this study, unlike the literature, DESs were blended with another polymer to obtain a solid film and used as a heterogeneous catalyst in GLC synthesis. DES-polymer interactions and membrane production have been extensively studied; however, there are no reports on the immobilization of KOH-based DES as an active heterogeneous catalytic membrane within a polymer for GLC synthesis.^23,24^ Although DESs have been used in homogeneous reactions and incorporated into membrane structures in pervaporation studies to participate in separation processes, we have not encountered any studies where they were used as a catalyst in the solution phase in pervaporation membrane reactor configurations.^25,26^ Therefore, while using DESs to catalyze the reaction in the solution phase, we also investigated a hybrid system in our study where the balance is directed toward product formation by removing the byproduct from the reaction using a separate PVA membrane.

This study aimed to synthesize GLC from DMC and glycerin under mild conditions (low temperature, no toxic solvent) by a functional catalytic membrane prepared in film form by adding DES produced from KOH and PEG to PVA. The catalytic membrane produced eliminates the catalyst separation process and reduces the cost of catalyst recovery. DES prepared from PEG and KOH is environmentally friendly. In this study, DES added to the PVA matrix acted as a heterogeneous catalyst. PVA/DES catalytic membranes were used as catalysts in the glycerin transesterification shown in Figure 1.

**Figure 1:**
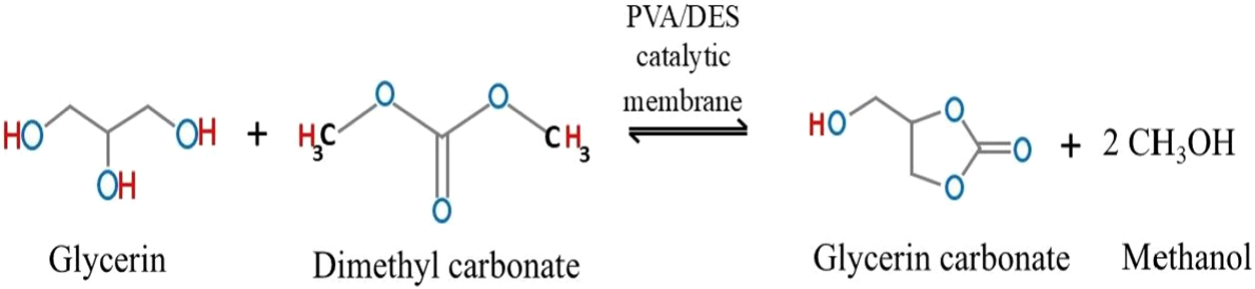
GLC synthesis by transesterification.

## 2 Materials and Methods

### 2.1 Materials

PVA (Mw ∼ 125,000), PEG-400, and DMC (for synthesis) were purchased from Sigma-Aldrich. KOH and Glycerin (anhydrous for synthesis) were obtained from Merck.

### 2.2 Preparation of KOH/PEG-400 DES

For DES production, certain amounts of KOH and PEG were mixed at 80 °C and 400 rpm for 1 h. KOH/PEG, which was obtained in a 1:2 molar ratio and has a dark color, was added as a catalyst to the PVA matrix.^18^

### 2.3 Preparation of PVA/DES Catalytic Membranes

Six wt % PVA was dissolved in distilled water at 90 °C for 2 h. The amounts of DES shown in Table 1 were mixed with the same amount of PVA solution, poured onto melamine plates, and left to dry. As shown in Figure 2, PVA/DES catalytic membranes were fabricated as films.

**Figure 2:**
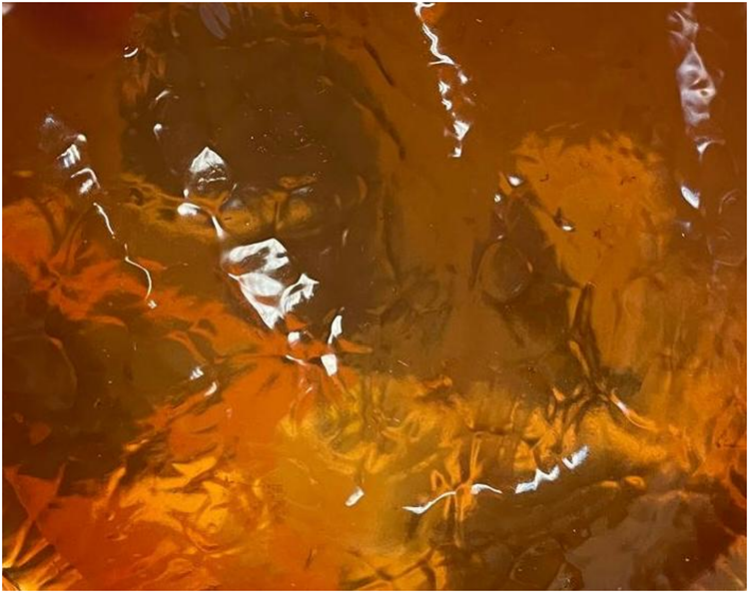
PVA/DES catalytic membrane.

**Table 1:** Content of DES Catalysts

KOH (g)	PEG-400 (g)	DES (catalyst) (g)	catalyst % for glycerin
0.1	1.43	1.53	11%
0.2	2.86	3.06	22%
0.3	4.28	4.58	33%

### 2.4 Batch Reactions with PVA/DES Catalytic Membranes

For each experiment, a 10.5 cm diameter catalytic membrane (containing the specified amounts of DES) was cut into small pieces measuring approximately 1 cm × 1 cm and added to the batch reactor. The estimated surface area of the catalytic membrane is 173.1 cm^2^, measured by calculating the surface area of both surfaces (excluding the surface area contributed by the membrane thickness). Reactions using the functional PVA/DES catalytic membrane were carried out in batch reactors at 11%, 22%, and 33% catalyst loadings; at 55, 70, and 85 °C temperatures; and with DMC/glycerin:1:1, 2:1, 3:1, 4:1, and 5:1 molar ratios. As shown in Figure 3, small pieces of PVA/DES catalytic membranes were put into the reactor to increase the catalyst surface area.

**Figure 3:**
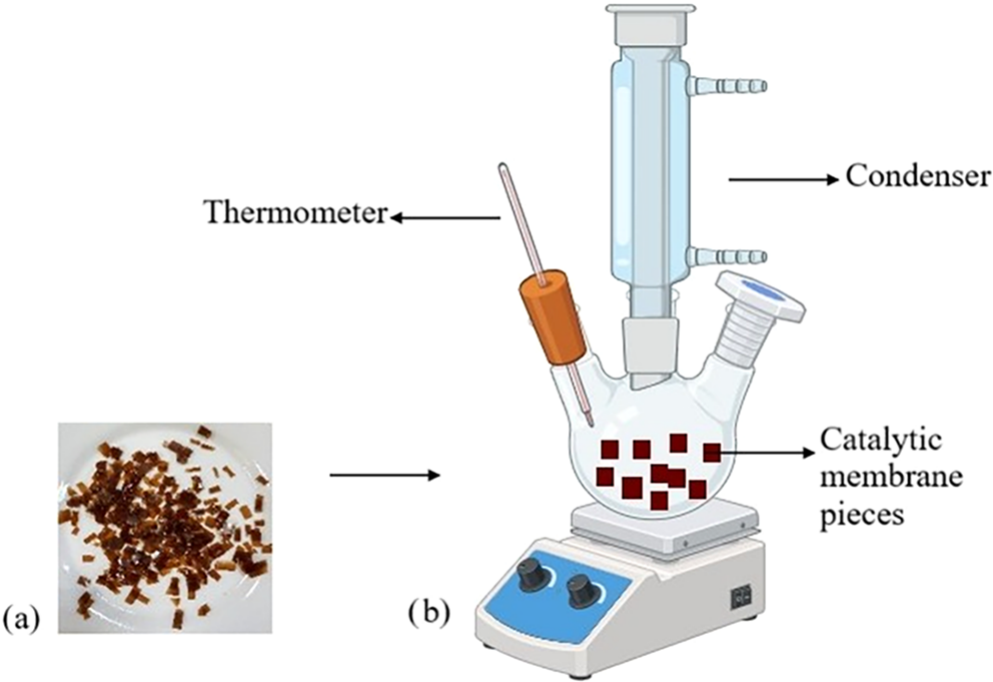
Reaction system; (a) Catalytic membrane pieces and (b) batch reactor system.

### 2.5 Membrane Reactor System

In the membrane reactor drawing shown in Figure 4, the membrane to be used for separation is placed in the cell. The reactant and catalyst are added to the cell. The reaction takes place on the membrane in the cell. With the vacuum applied from the bottom of the hydrophilic membrane, the reaction byproduct (water or methanol) is transported through the membrane, exits from the bottom as vapor, where it condenses in liquid nitrogen traps and is collected in liquid form.^27,28^ The system is also called a pervaporation membrane reactor. The reaction cell in the membrane reactor has a volume of 250 mL and a length of 10 cm. In this cell, a PVA membrane with a diameter of 5 cm was used as the separation membrane. The effective area of the PVA membrane is 19.6 cm^2^.

**Figure 4:**
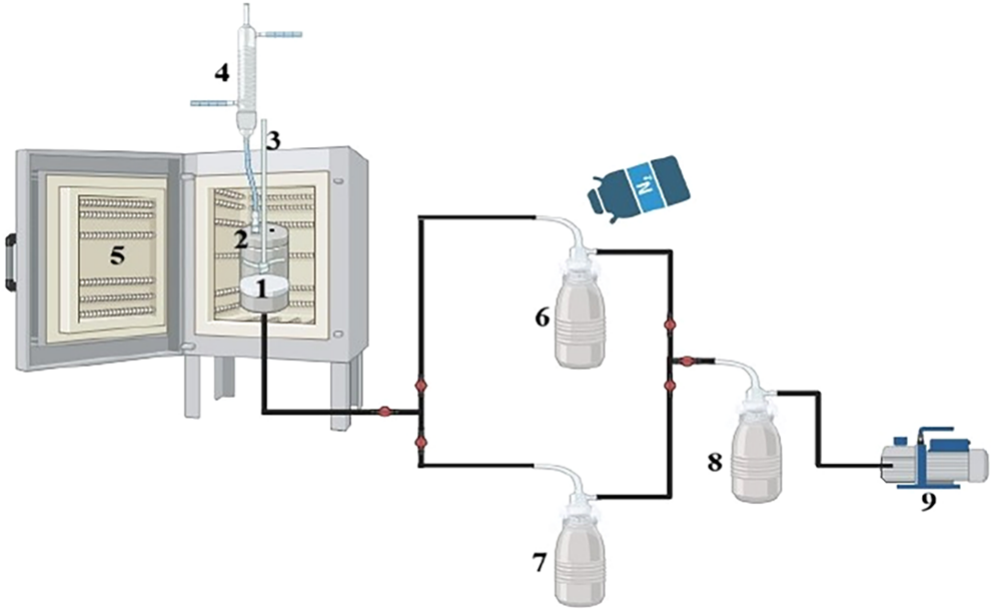
Membrane reactor system: (1) PVA Membrane, (2) Reactor cell, (3) mechanical stirrer, (4) Condenser, (5) Oven, (6–8) Cold Traps, (9) Vacuum pump.

### 2.6 Quantitative Analysis of GLC

The amount of GLC (mol) from the reaction was calculated using gas chromatography (GC). Helium is the carrier gas in the GC with an FID detector and a DB-wax column, and the detector temperature was set to 280 °C. The GLC yield was calculated by the following eq 1 ^29^

1 $$\mathrm{GLC}\;y\mathrm{ield}\;\left(\%\right)=\frac{p\mathrm{roduced}\;\mathrm{GLC}\;\left(\mathrm{mol}\right)}{i\mathrm{nitial}\;\mathrm{glycerin}\;\left(\mathrm{mol}\right)}\times 100$$

## 3 Results and Discussion

### 3.1 Characterization of PVA/DES Catalytic Membranes

Characterization results of PVA/DES catalytic membranes are given in the Supporting Information.

### 3.2 Batch Reactions Using PVA/DES Catalytic Membranes

The synthesis of GLC from glycerin and DMC was carried out with PVA/DES catalytic membranes under the conditions mentioned in Section 2.4, and the influence of reaction parameters such as catalyst amount, reaction temperature, DMC/glycerin initial molar ratio, and time on GLC yield was investigated. The impact of each parameter is analyzed separately.

#### 3.2.1 Optimal Reaction Time Determination

To determine the optimum reaction time, the reaction was carried out for up to 6 h. As shown in Figure 5a, the maximum GLC yield was reached at 4 h. Reaction conditions are 33% catalyst amount, at 85 °C reaction temperature, and DMC/glycerin at a ratio of 3:1. The reaction yield reached a maximum of 65.4% at 4 h and then decreased to 55.8% at 5 h. Although the GLC yield started to increase again at the sixth hour (60.3%), the optimum reaction time was determined to be 4 h, considering time and energy savings. The decomposition of the major product GLC to glycidol due to decarboxylation may have led to a decline in GLC yield.^10,30^ The presence of glycidol in the reaction was confirmed by Fourier-transform infrared (FTIR) analysis.

**Figure 5:**
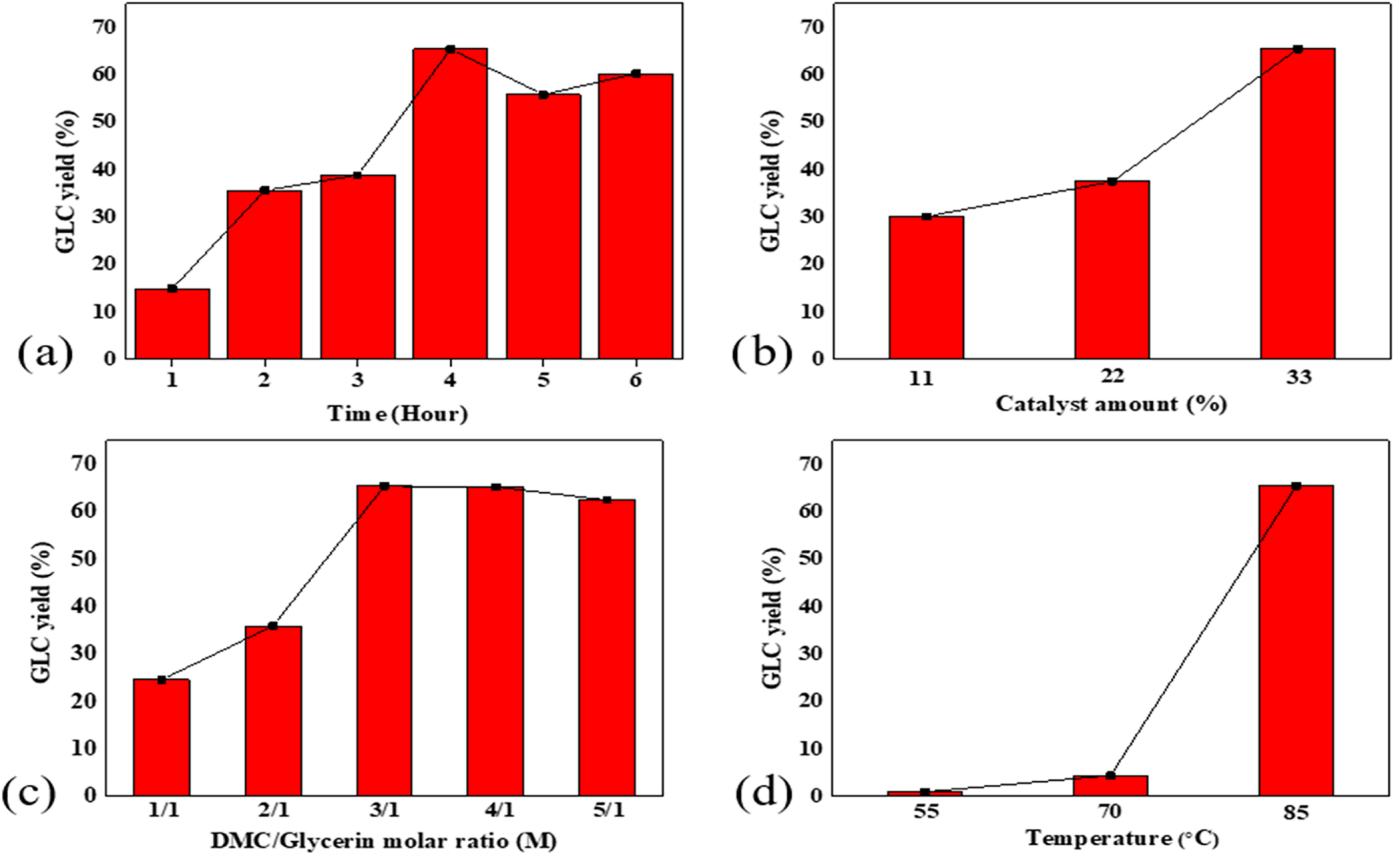
Effects of reaction parameters on reaction yield; (a) Time, (b) Catalyst amount, (c) DMC/glycerin molar ratio, (d) Temperature.

#### 3.2.2 Effect of Catalyst Amount on GLC Yield

In catalytic reactions, the amount of catalyst is an important reaction parameter. GLC was synthesized at 85 °C using catalytic membranes containing 11%, 22%, and 33% DES at a molar ratio of M:3/1 for 4 h. As depicted in Figure 5b, the GLC yield increased from 30.1% at 11% DES to 65.4% at 33% DES. Base catalysts are often used in GLC synthesis because they more easily activate glycerin to react.^31^ As the proportion of DES in the catalyst increases, the amount of catalytically active basic sites also increases, due to the rise in KOH. Since catalytic activity increases with the number of catalytically active sites per unit, as the amount of DES increased, the GLC yield also increased.^32,33^

#### 3.2.3 Effect of Initial Molar Ratio of DMC/Glycerin

In synthesizing carbonate from DMC and glycerin, which is a reversible equilibrium reaction, increasing the amount of DMC can enhance the formation of GLC by shifting the reaction equilibrium toward GLC production.^34^ Figure 5c presents the effect of different DMC/Glycerin molar ratios on GLC yield for reaction conditions of 85 °C, 33% catalyst amount, and 4 h reaction time. When the DMC/glycerin molar ratio was increased from 1:1 to 3:1, the GLC yield increased significantly from 24.6% to 65.4%. Subsequently, the reaction yield remained constant at 4:1 and decreased to 62.5% at M:5/1. Excess DMC decreased the yield by reducing the concentration of glycerin in contact with the catalytic membrane.^35,36^

#### 3.2.4 Effect of Reaction Temperature on GLC Yield

The reaction temperature is one of the critical parameters that can contribute to the reaction yield by directly affecting the reaction rates in catalytic reactions.^37^ The effect of three different reaction temperatures (55, 70, and 85 °C) on GLC yield at 33% catalyst amount, M:3/1 molar ratio, and 4 h reaction conditions, is indicated in Figure 5d. At 55 °C, no significant GLC yield could be obtained. When the temperature was increased from 70 to 85 °C, however, the GLC yield increased dramatically from 4.4% to 65.4%. The increase from 55 to 70 °C did not considerably affect the reaction yield. Based on the Arrhenius equation, at a reaction temperature of 85 °C, the velocity and collisions of the reactants increased, thereby enhancing the possibility of product formation.^38^ In addition, the decrease in viscosity and increase in solubility of glycerin at high temperatures decreased the mass transfer resistance and facilitated the reaction of glycerin with DMC.^39^

### 3.3 Comparison of Batch Operation with a Membrane Reactor

The reaction, carried out in the batch reactor at 85 °C, with a 33% catalyst ratio, a 3:1 molar ratio, and a 3 h reaction time, was also carried out in the membrane reactor under the same conditions. Figure 6 shows the comparison of GLC synthesis in the reactors in terms of yield. In the membrane reactor system shown in Figure 4, the prepared DES catalyst was put into the reactor, and a GA-cross-linked pure PVA membrane was placed in the membrane separation section. In addition, the results obtained are compared with the freely used DES catalyst in the batch reactor under the same conditions. In the comparison, the membrane reactor gave the highest GLC yield of 74.0% under the same conditions. With the vacuum applied to the pure PVA membrane in the membrane reactor, the reaction byproduct methanol was removed from the reaction medium, and the reaction equilibrium shifted toward product formation, thus increasing the GLC yield.^40,41^

**Figure 6:**
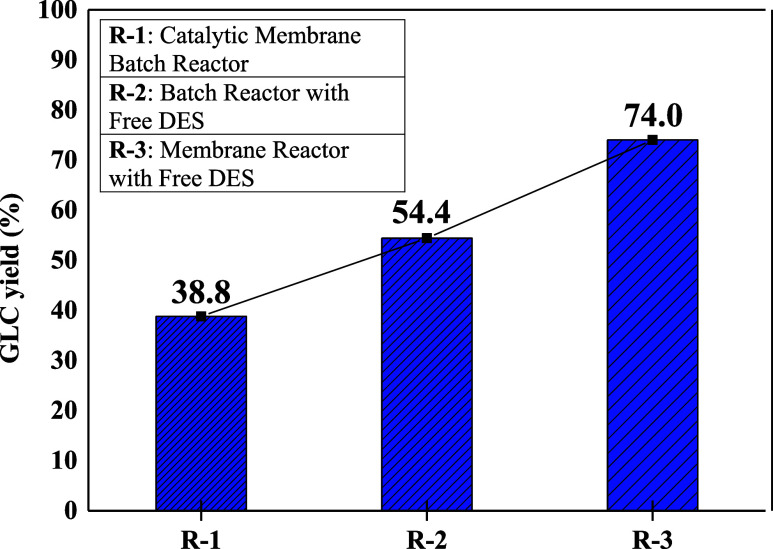
Comparison of GLC synthesis by different methods.

### 3.4 Comparison of Batch and Membrane Reactor Experiments with Recent Literature Results

For GLC synthesis, the basicity of catalysts depends on many factors, such as the type, accessibility, and strength of basic centers.^42^ According to Table 2, Higher reaction yields (80–95%) were achieved in studies using highly basic catalysts such as (Volcanic lava ash, Mg–Fe mixed metal oxide, CaO-CeO_2_, NaTNT). Based on the reaction yield results, NaOH, DES, and PVA/DES catalytic membranes have shown moderate basic activity (62.84%, 54.4–74.0%, 65.4%). Some carbon derivatives (alginate-derived carbon, biochar, coffee ground ash) produced lower reaction yields due to the low strength of phenolic and carboxylate groups on the surface.^43^ Therefore, methods such as pyrolysis, calcination, modification, and base treatment are required for use in basic-catalyzed reactions.^44,45^ Other studies in the literature on GLC production using catalytic membranes have attracted attention due to their high yield and conversion values.^22,46,47^ Although the maximum GLC yields obtained in this study, 65.4% and 74.0%, are low compared to other catalytic membrane studies where high yields were achieved, the catalytic membrane is more environmentally friendly and safer, as it does not contain any severely corrosive materials in terms of all the materials it contains (DES and PVA).^48^ Additionally, regular methanol removal using a pervaporation membrane reactor system disrupted the reaction equilibrium, promoting a shift in equilibrium toward product formation. This ensured a more sustainable GLC synthesis.^49,50^ The preparation of DES and PVA/DES catalytic membranes does not require the use of the mentioned methods and has a very simple and low-energy-consuming preparation procedure. Additionally, by selective removal of byproducts, the pervaporation membrane reactor application is a highly effective and environmentally friendly method for increasing reaction yield.^51^

**Table 2:** Comparison of This Study with Recent Literature Results

reaction	conditions	catalyst	yield (%)	refs
glycerin and DMC	75 °C, cat.: %5 (wt), M = 3:1, 30 min	pyrolized sodium alginate	57	^52^
glycerin and DMC	75 °C, cat.: %5 (wt), M = 3:1, 90 min.	volcanic lava ash	91	^39^
glycerin and DMC	75 °C, cat.: %4 (wt), M = 4:1, 60 min.	spent coffee grounds and mussel shells	49.7	^11^
glycerin and DMC	90 °C, cat.: %10 (wt), M = 3:1, 60 min.	CaO–CeO_2_	94.05	^53^
glycerin and DMC	90 °C, cat.: %5 (wt), M = 5:1, 90 min.	NaTNT (sodium titanate nanotubes)	92.6	^42^
glycerin and DMC	110 °C, cat.: %5 (wt), M = 5:1, 20 min.	sunflower stalk-derived biochars	34.9	^54^
glycerin and DMC	90 °C, cat.: 0.2 g M = 4:1, 150 min.	Mg–Fe mixed metal oxides	83.2	^55^
glycerin and DMC	120 °C, cat.: 3% (wt), M = 4:1, 60 min.	NaOH	62.84	^56^
glycerin and DMC (batch reactor)	85 °C, cat.: 33% (wt), M = 3:1, 240 min.	PVA/DES catalytic membrane	65.4	this work
glycerin and DMC (batch reactor)	85 °C, cat.: 33% (wt), M = 3:1, 180 min.	PVA/DES catalytic membrane	38.8	this work
glycerin and DMC (batch reactor)	85 °C, cat.: 33% (wt), M = 3:1, 180 min.	DES	54.4	this work
glycerin and DMC (membrane reactor)	85 °C, cat.: 33% (wt), M = 3:1, 180 min.	DES	74.0	this work
glycerin and DMC	70 °C, cat.: 3% (wt), M = 3:1	chitosan-eggshell catalytic membrane	77 (conversion)	^47^
glycerin and DMC	75 °C, cat.: 1.6% (wt), M = 3:1, 120 min.	PVA/NaOH catalytic membrane	93.03	^46^
glycerin and DMC	75 °C, cat.: 2.7% (wt), M = 3:1, 180 min.	PVA/PVP/sodium methoxide catalytic membrane	99.7	^22^

### 3.5 RSM Study with Central Composite Design

The RSM study conducted a statistical analysis of the batch reaction results and compared the effect of reaction parameters on the GLC yield. The RSM study was carried out in the DESIGN EXPERT program for two variables (catalyst amount and DMC/Glycerin) by selecting the central composite design. The variables to be analyzed and the limit values entered are displayed in Table 3 with codes A (catalyst amount) and B (DMC/glycerin molar ratio). When the values of the design variables to be examined are entered into the system, the central composite design proposes 13 experiments, as indicated in Table 4. Points outside the design limits ((6.4, 37.5) and (0.6, 3.4)) were also examined for the 13 experiments performed.

**Table 3:** Design Variables Coded A and B

	name	unit	low	high	-α	+α
A	catalyst amount	%	11	33	6.4	37.5
B	DMC/glycerin	M	1	3	0.6	3.4

**Table 4:** 13 Experiments Offered by Central Composite Design

run	catalyst amount (%)	DMC/glycerin (M)	response (yield %)
1	11	3	30.1
2	22	2	34.9
3	22	2	34.9
4	33	3	65.4
5	22	2	34.9
6	11	1	19.7
7	6.4	2	23.6
8	22	3.4	38.3
9	22	2	34.9
10	22	0.6	11.2
11	33	1	24.6
12	37.5	2	43.4
13	22	2	34.9

According to the compatibility of the reaction results, the model suggested by the program is the 2FI model, as shown in Table 5. The 2FI model is proposed as the most appropriate model for the data. Table 6 shows that the 2FI model has the highest predicted *R* ^2^, confirming this suggestion from the data.

**Table 5:** Sequential Model Sum of Squares

source	sum of squares	df	mean square	*F*-value	*P*-value	
mean vs total	14,276.05	1	14,276.05			
linear vs mean	1583.27	2	791.64	17.99	0.0005	
**2FI vs linear**	**231.04**	**1**	**231.04**	**9.95**	**0.0117**	**suggested**
quadratic vs 2FI	101.77	2	50.89	3.32	0.0967	
cubic vs quadratic	39.32	2	19.66	1.45	0.3190	aliased
residual	67.86	5	13.57			
total	16,299.32	13	1253.79			

**Table 6:** Model Summary Statistics

source	Std dev.	*R* ^2^	adjusted *R* ^2^	predicted *R* ^2^	press	
linear	6.63	0.7825	0.7390	0.5279	955.19	
**2FI**	**4.82**	**0.8967**	**0.8623**	**0.7252**	**556.00**	**suggested**
quadratic	3.91	0.9470	0.9092	0.6233	762.18	
cubic	3.68	0.9665	0.9195	–1,1466	4343.12	aliased
						

The final equation for the design variables and their binary interactions based on the 2FI model is shown below.

response(yield):33.14+8.53×A+11.19×B+7.60×AB

In Table 7, which shows the variance analysis for the 2FI model, the *p*-value is <0.0001, indicating that the model is significant. When the effects of the model variables on the reaction yield are compared, the molar ratio term (*B*) has the highest effect, with the lowest *p*-value (0.0001) and the highest *F*-value (43.65).

**Table 7:** ANOVA for 2FI Model

source	sum of squares	df	mean square	*F*-value	*p*-value
model	1814.31	3	604.77	26.05	<0.0001
A	581.43	1	581.43	25.04	0.0007
B	1001.84	1	1001.84	43.15	0.0001
AB	231.04	1	231.04	9.95	0.0117
residual	208.96	9	23.22		
lack of fit	208.96	5	41.79		
pure error	0.0000	4	0.0000		
cor total	2023.27	12			

According to the fit statistics in Table 8, the difference between the predicted *R* ^2^ (0.7252) and adjusted *R* ^2^ (0.8623) is less than 0.2, showing that the model is acceptable. Also, adequate precision greater than 4 (16.1411) suggests that the model can be used in the design area. Figure 7 presents the residual plots of the proposed model. Residuals indicate the difference between predicted and actual values and are expected to be close to 0.^57^ In Figure 7a, the data distribution is mostly close to zero and to the red line, showing that the data are distributed close to a normal distribution, with the exception of run 8, and most of the residuals can be considered insignificant.^58,59^ In Figure 7b,c, most of the data are randomly distributed around zero except run 8, emphasizing the accuracy and adequacy of the model assumption.^60,61^ Run 8 should be repeated to increase the adequacy of the model.

**Figure 7:**
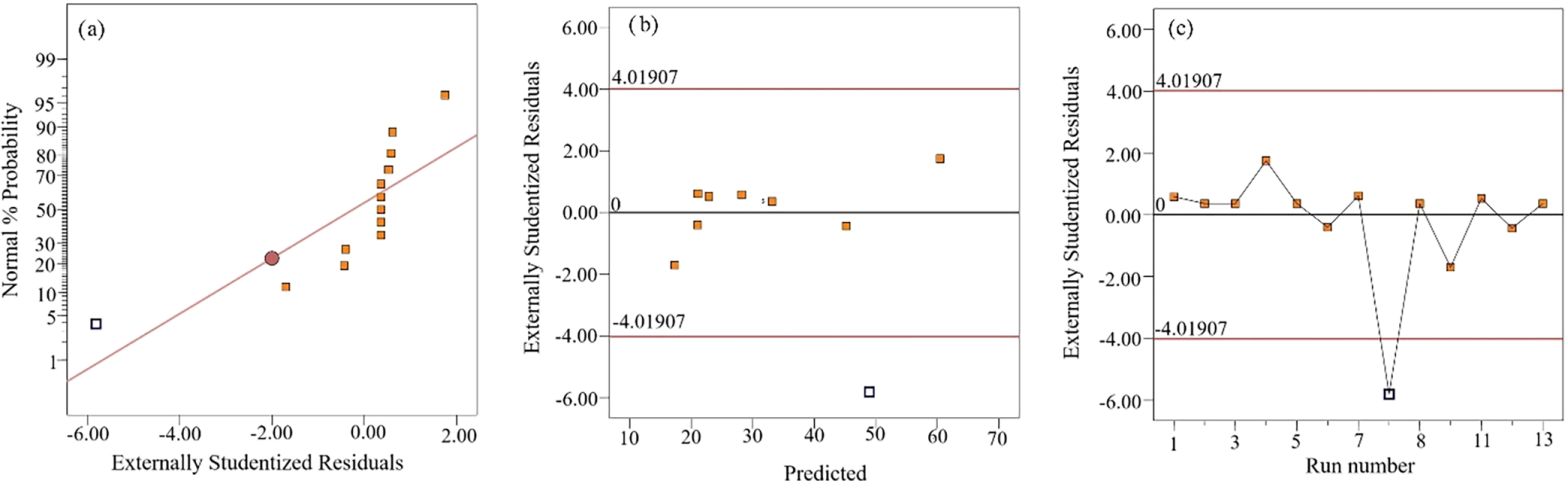
Residual plots of model, (a) Normal plots of residuals, (b) Residual vs Predicted, (c) Residuals vs Run number.

**Table 8:** Fit Statistics

Std. Dev.	4.82	*R* ^2^	0.8967
mean	33.14	adjusted *R* ^2^	0.8623
C. V. %	14.54	predicted *R* ^2^	0.7252
		Adeq precision	16.1411

Figure 8 shows the closeness between the actual yield results and the results predicted by the model. Here, it is observed that the measured value from run 8 (38.3%) and the predicted value by the model (49%) do not closely match. Therefore, run 8 was repeated, and a 38.3% GLC yield was again obtained.

**Figure 8:**
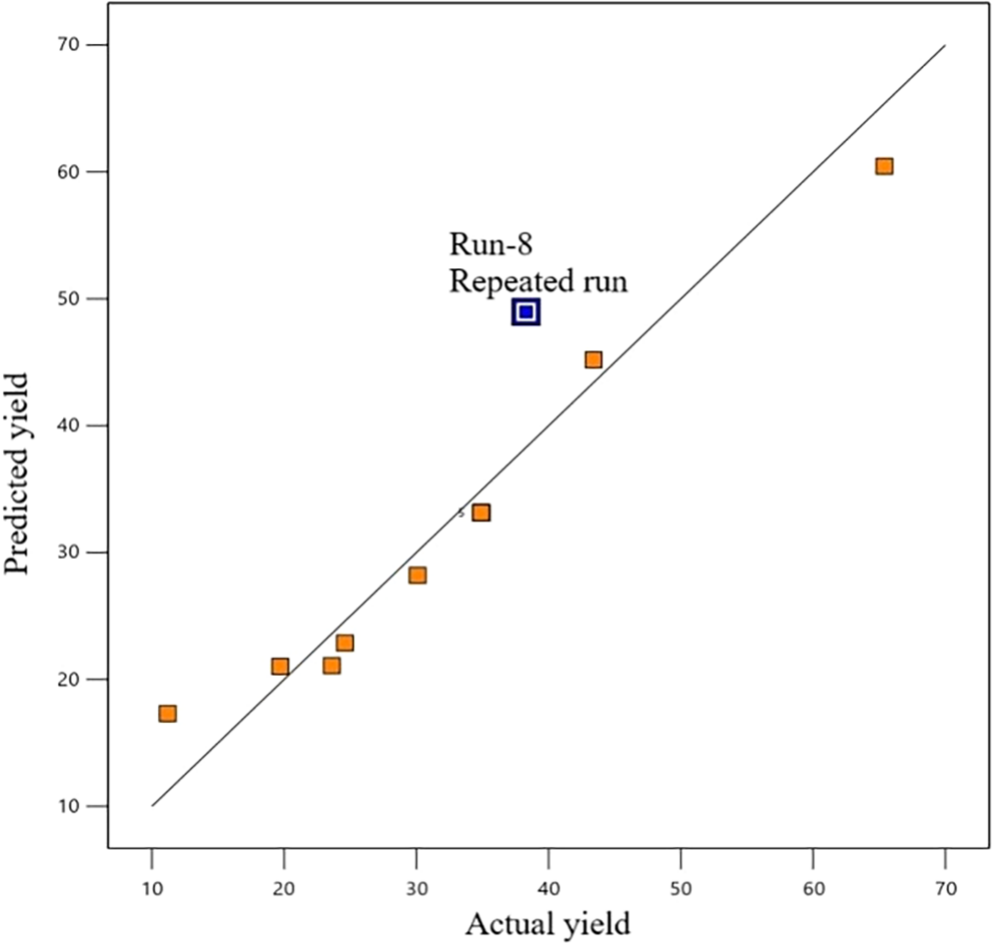
Predicted and actual yield values.

The perturbation plot given in Figure 9 shows that the investigated parameters contribute positively, with a linear increase, to the GLC yield. It also shows the comparative effects of model variables on the response at a given reference point. The 3D plot in Figure 10 shows the effect of the change in model variables on the GLC yield using colors. A steady increase in GLC yield was observed with increasing DMC/Glycerin molar ratio from M:1/1 to M:3/1 and catalyst amount from 11% to 22%.

**Figure 9:**
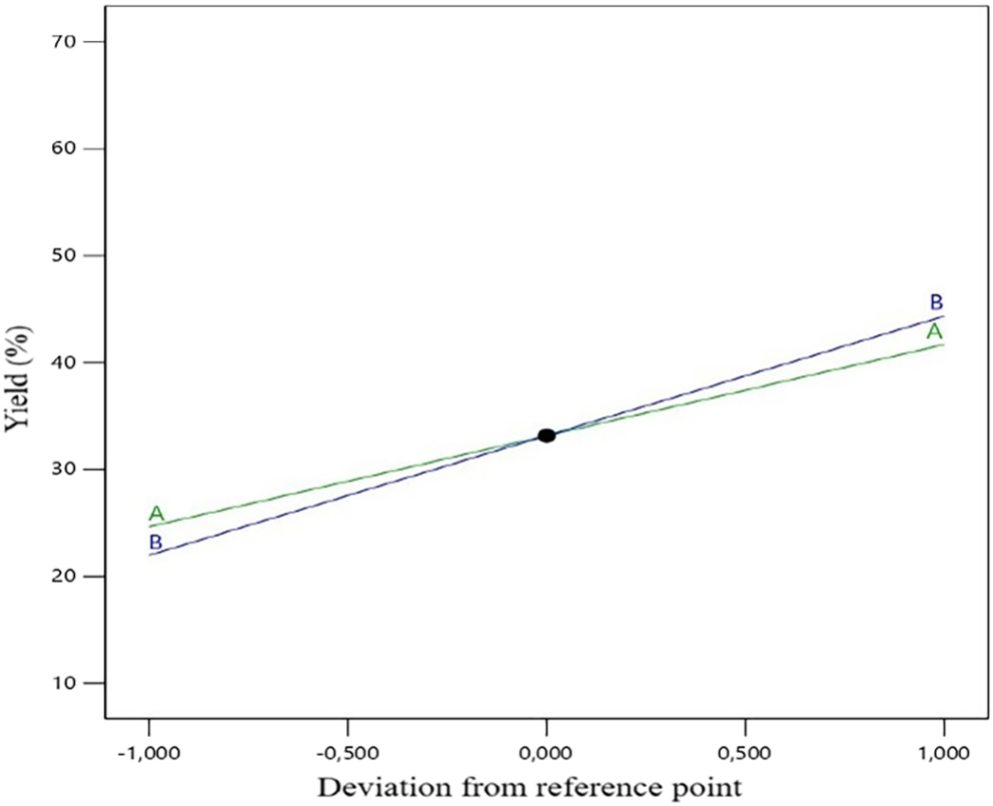
Perturbation plot of model variables.

**Figure 10:**
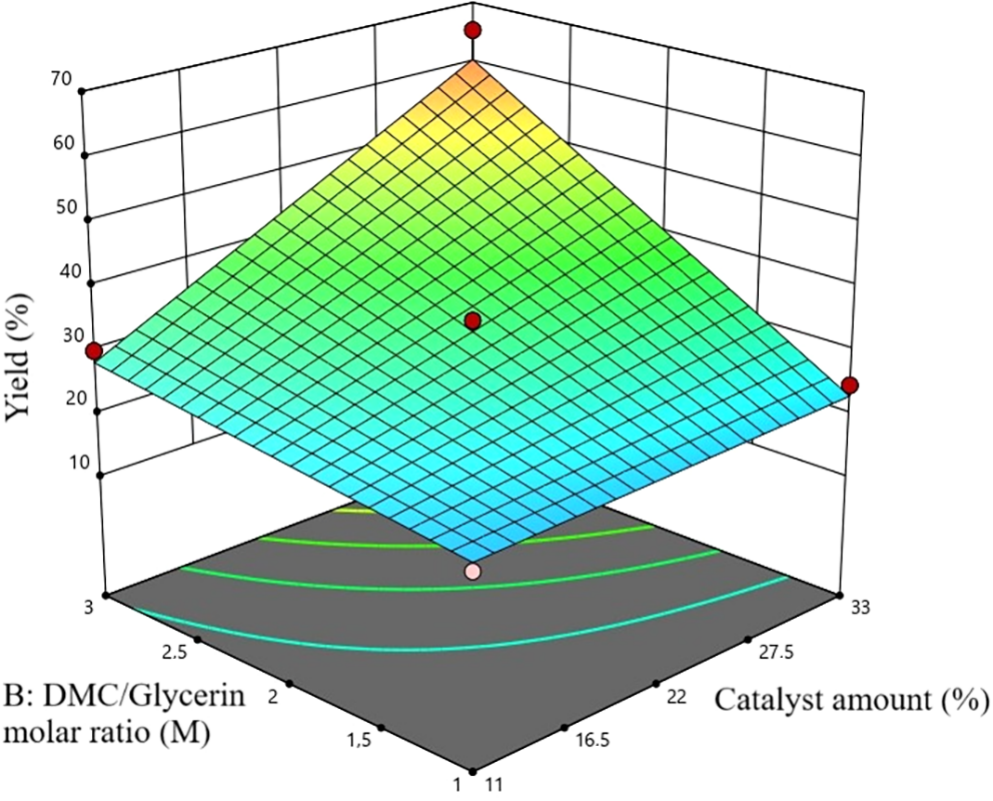
3D plot.

## 4 Conclusions

GLC, a promising and value-added product from the perspective of clean energy use, was synthesized under atmospheric pressure and without toxic solvents with a PVA-supported DES catalyzed heterogeneous catalytic membrane. Green DES prepared from KOH and PEG in homogeneous form is combined with the PVA matrix to obtain a heterogeneous catalyst. The heterogeneous catalytic membrane was easily recovered after the reaction. In FTIR analysis of the PVA/DES catalytic membrane before the reaction, DES loading was confirmed by CH_2_ stretching and bending vibrations at the peaks at 1453 cm^–1^ and 2870 cm^–1^, respectively. In the FTIR analysis after the reaction, the peak at 1782 cm^–1^ corresponds to the presence of cyclic carbonate, which is attributed to the GLC formed in the membrane structure. The absorbance peak observed at 1048 cm^–1^, belonging to methanol, showed that the catalytic membrane was able to absorb this byproduct. Thermogravimetric analysis (TGA) analysis showed that the DES catalyst added to the PVA matrix increased the thermal resistance. The high hydrophilicity of the PVA/DES catalytic membrane was confirmed by contact angle measurements (16.9, 14.2, 24.3°). In the RSM study with central composite design, DMC/glycerin molar ratio (*F*-value: 43.15) has a greater effect on the GLC yield than the amount of catalyst (*F*-value: 25.04). A maximum GLC yield of 65.4% was achieved in batch reactions with PVA/DES catalytic membrane under the specified reaction conditions (85 °C, 33% catalyst amount, DMC/glycerin: 3:1, 4 h). Additionally, the optimum reaction time was reduced by increasing the reaction yield to 74.0% using a 3:1 DMC/glycerin molar ratio, at 3 h, 85 °C, and 33% catalyst amount in the membrane reactor. In the membrane reactor, the byproduct was removed from the reaction medium with the aid of a separator using a PVA membrane and vacuum, contributing to the reaction yield. The active role of DESs that are catalytically promising and environmentally friendly, gaining heterogeneous properties in reactions, is a significant progress toward the development of greener catalytic systems.

## Supplementary Material



## Notes

FTIR and TGA analysis of PVA/DES catalytic membranes; contact angle measurements of PVA/DES catalytic membranes; and FTIR analysis of PVA/DES membranes before and after the reaction (PDF)
