# Supplementary material for: Synthesis of Glycerin Carbonate by an Environmentally Friendly Novel Catalytic Membrane of Poly(vinyl alcohol)/Deep Eutectic Solvent
Source: ACS Omega. 2026 Mar 4;11(10):16017–25. doi: 10.1021/acsomega.5c10125 (PMC13000604; doi:10.1021/acsomega.5c10125)
Supplement: Supplementary file 1 [file ao5c10125_si_001.pdf]

Supporting Information

Synthesis of Glycerin Carbonate by an Environmentally Friendly Novel Catalytic Membrane Polyvinyl alcohol/Deep eutectic solvent

Guler Hasirci[a], *Nilufer Durmaz Hilmioglu*[a]*

[a]Chemical Engineering Department of Kocaeli University, İzmit, Kocaeli, Turkey

* Corresponding author: Nilufer Hilmioglu

*E-mail address: niluferh@kocaeli.edu.tr

1. FTIR Characterization of PVA/DES Catalytic Membranes

Absorbance scanning was performed from 650 to 4000 cm^{-1} in FTIR analysis. Fig. S1 depicts the FTIR spectra of PVA/DES catalytic membranes and compares them with pure PVA and PEG. In the PVA/DES catalytic membrane, the O-H peak of PVA at 3294.48 cm^{-1} shifted to the absorbance peak at 3347 cm^{-1} due to the addition of PEG to the structure. [1] The shift in the -OH band indicates the hydrogen bond interaction between the -OH groups of DES and the -OH groups of PVA, while also indicating a strong molecular interaction between PVA and DES.[2,3] In addition to hydrogen bond interactions between PVA and DES, ion-dipole interactions originating from K^+ cations may also be involved. Ion-dipole interactions are attractive forces that occur between fully charged ions and partially charged dipole groups. [4] Consistent with similar examples in the literature, the ion-dipole interaction formed between K^+ cations in DES and OH groups in PVA is supported by the decrease in the -OH band vibration and its shift to higher wavelengths in PVA/DES samples. [5,6] The lack of OH band vibration in pure DES may be due to the elimination of free OH groups as a result of hydrogen bond interactions between KOH and PEG.[7] The peaks at 2870 and 1453 cm^{-1} originate from PEG and occur due to CH_2 stretching and bending vibrations, respectively. [8] The peak at 1087 cm^{-1} of the C-O stretching vibration of PVA shifted to 1097 cm^{-1} in the PVA/DES structure. [9] This shift is due to the interaction between PVA and DES and their good mixing in terms of phase. [10] In addition, the intensity of the peaks at 1097 cm^{-1} increased as the amount of PEG in the PVA structure increased. [11]

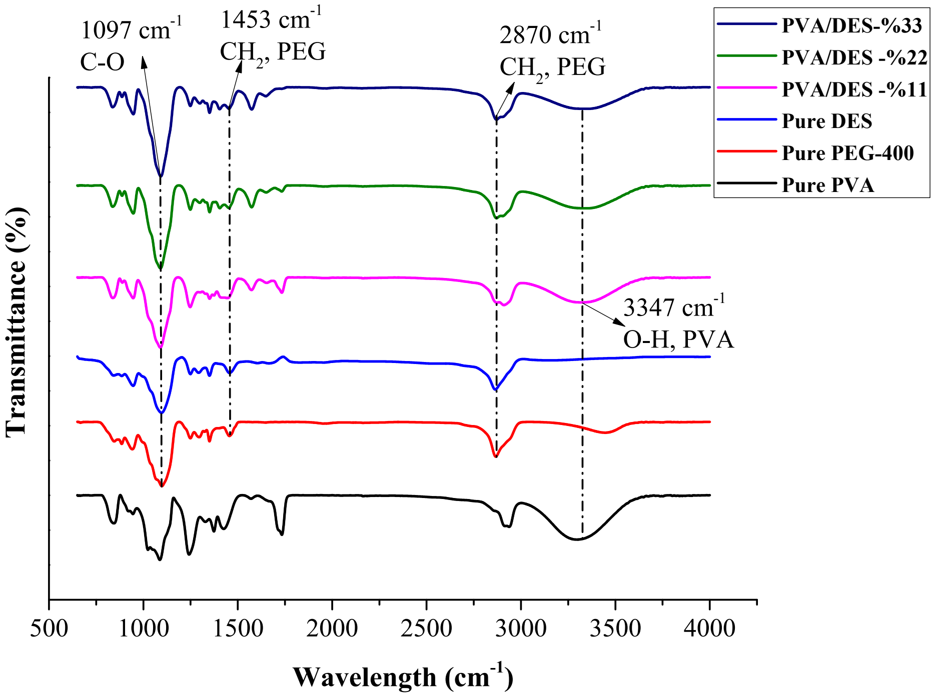

Fig. S1 FTIR of PVA/DES catalytic membranes

2. TGA analysis of PVA/DES catalytic membranes

The temperature-dependent mass losses and thermal stability of the produced catalytic membranes were investigated by TGA. Thermogravimetric analysis (TGA) of PVA, PEG, and PVA/DES, catalytic membranes is presented in Fig. S2. Only one degradation region between 250 °C and 390 °C was observed for pure PEG 400. [12] In the TGA analysis of pure PVA, three different degradation regions were observed. While the degradation in the 1st region between 100-270°C is due to the separation of water molecules from the solution, the decomposition in the region between 270- 420°C is attributed to the elimination of water molecules bound to the polymer by OH bonds. The polymer was decomposed in the third region (420-480°C). Similar to PVA, PVA/DES catalytic membranes show three different degradation zones. The mass loss of about 5-7% between 50 and 100°C is the removal of physically absorbed water molecules from the structure. [3] The major mass loss (80 %) occurred between 230 and 460 °C. PEG-400 has shown lower thermal stability compared to PVA. DES reduced the thermal stability of the PVA/DES structure by lowering the initial decomposition temperatures compared to PVA. [13]

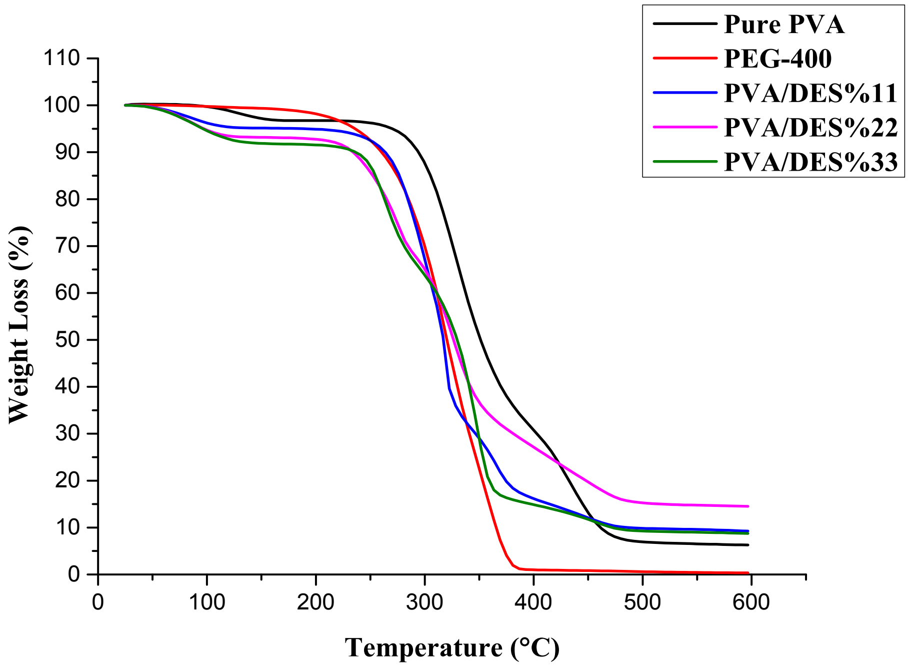

Fig. S2 TGA of PEG, PVA polymers, and PVA/DES catalytic membranes

3. Contact angle measurements of PVA/DES catalytic membranes

The contact angle measurements shown in Fig. S3 were used to determine the hydrophilicity of PVA/DES catalytic membranes. The very low contact angles for all catalyst ratios indicate that the membranes have high hydrophilicity. [14] The high hydrophilicity and polarity of the catalytic membrane may lead to a better interaction between glycerin and the membrane.[15] Uncrosslinked PVA and PEG are highly hydrophilic polymers. A slight decrease in the contact angle was observed as the catalyst content increased from 11% to 22%. This can be attributed to the increase in the amount of PEG and KOH in the structure.[16, 17] However, an increase in contact angle was observed at 33% catalyst ratio. The increment here can be explained by the plasticizing nature of PEG. [18]

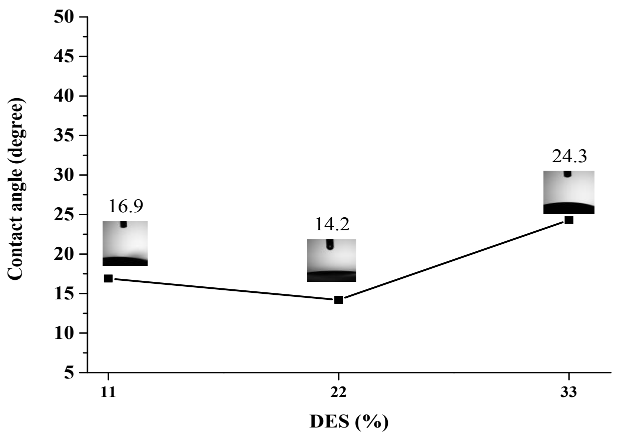

Fig. S3 Contact angles of PVA/DES membranes.

3. FTIR analysis of PVA/DES membranes before and after the reaction

FTIR analysis of the same PVA/DES catalytic membrane piece before and after the reaction is presented in Fig. S4. The peak observed at 1782 cm^{-1} occurs from the C=O carbonyl stretching due to cyclic carbonate, confirming the presence of GLC. [19, 20] The vibration band at 1178 cm^{-1} is the C-O stretching originating from the 2-hydroxy ethyl chain, supporting the presence of GLC.[21] The peaks associated with GLC confirm that the reaction occurs on the membrane. The 1048 cm^{-1} peak, which is associated with methanol as a by-product of the reaction, indicates that the catalytic membrane retains methanol.[22] The peak at 774 cm^{-1} is attributed to the epoxy linkage of glycidol, another product formed in the reaction. [23]

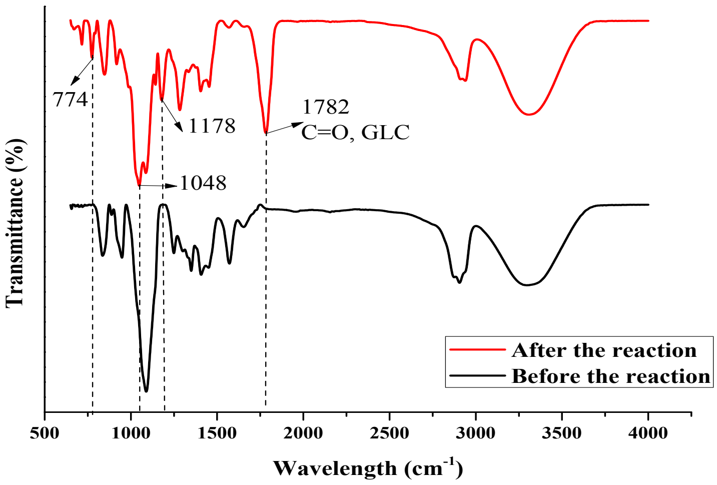

Fig. S4 FTIR of PVA/DES-33% catalytic membrane before and after the reaction

REFERENCES

(1) Prajapati, G. K.; Gupta, P. N. Comparative Study of the Electrical and Dielectric Properties of PVAPEGAl2O3MI (M=Na, K, Ag) Complex Polymer Electrolytes. *Phys. B Condens. Matter* **2011**, *406* (15–16), 3108–3113. https://doi.org/10.1016/j.physb.2011.05.019.

(2) Alibwaini, Y. A.; Hemeda, O. M.; El-Shater, R.; Sharshar, T.; Ashour, A. H.; Ajlouni, A. W.; Arrasheed, E. A.; Henaish, A. M. A. Synthesis, Characterizations, Optical and Photoluminescence Properties of Polymer Blend PVA/PEG Films Doped Eosin Y (EY) Dye. *Opt. Mater. (Amst).* **2021**, *111* (August 2020), 110600. https://doi.org/10.1016/j.optmat.2020.110600.

(3) Falqi, F. H.; Bin-Dahman, O. A.; Hussain, M.; Al-Harthi, M. A. Preparation of Miscible PVA/PEG Blends and Effect of Graphene Concentration on Thermal, Crystallization, Morphological, and Mechanical Properties of PVA/PEG (10wt%) Blend. *Int. J. Polym. Sci.* **2018**, *2018*. https://doi.org/10.1155/2018/8527693.

(4) Sippel, K. H.; Quiocho, F. A. Ion – Dipole Interactions and Their Functions in Proteins. **2015**, *24*, 1040–1046. https://doi.org/10.1002/pro.2685.

(5) Abdullah, O. G.; Aziz, S. B.; Rasheed, M. A. Structural and Optical Characterization of PVA:KMnO4 Based Solid Polymer Electrolyte. *Results Phys.* **2016**, *6*, 1103–1108. https://doi.org/10.1016/j.rinp.2016.11.050.

(6) Wahab, N. A.; Saadiah, M. A.; Ghazali, N. M.; Aoki, K.; Nagao, Y.; Mazuki, N. F.; Samsudin, A. S. Potassium Ion Coordination and Ionic Transport in Alginate–PVA Polymer Electrolyte. *Mater. Sci. Eng. B* **2025**, *322* (July), 118644. https://doi.org/10.1016/j.mseb.2025.118644.

(7) Chen, W.; Bai, X.; Xue, Z. The Formation and Physicochemical Properties of PEGylated Deep Eutectic Solvents. **2019**, 8804–8810. https://doi.org/10.1039/c9nj02196e.

(8) Sajjan, A. M.; Naik, M. L.; Kulkarni, A. S.; Fazal-E-Habiba Rudgi, U.; M, A.; Shirnalli, G. G.; A, S.; Kalahal, P. B. Preparation and Characterization of PVA-Ge/PEG-400 Biodegradable Plastic Blend Films for Packaging Applications. *Chem. Data Collect.* **2020**, *26*, 100338. https://doi.org/10.1016/j.cdc.2020.100338.

(9) Hemati, H.; Haghiralsadat, F.; Hemati, M.; Sargazi, G.; Razi, N. Design and Evaluation

of Liposomal Sulforaphane-Loaded Polyvinyl Alcohol/Polyethylene Glycol (PVA/PEG) Hydrogels as a Novel Drug Delivery System for Wound Healing. *Gels* **2023**, *9* (9). https://doi.org/10.3390/gels9090748.

(10) Sadiq, M.; Khan, M. A.; Hasan Raza, M. M.; Aalam, S. M.; Zulfequar, M.; Ali, J. Enhancement of Electrochemical Stability Window and Electrical Properties of CNT-Based PVA-PEG Polymer Blend Composites. *ACS Omega* **2022**, *7* (44), 40116–40131. https://doi.org/10.1021/acsomega.2c04933.

(11) Ali, Z. I.; Eisa, W. H. Characterization of Electron Beam Irradiated Poly Vinyl Alcohol/Poly Ethylene Glycol Blends. *J. Sci. Res.* **2013**, *6* (1), 29–42. https://doi.org/10.3329/jsr.v6i1.13071.

(12) Tien Nguyen, G.; Truong, T. A. N.; Duy Dat, N.; Phan, T. A. D.; Bui, T. H. Polyethylene Glycol Confined in SiO2-Modified Expanded Graphite as Novel Form-Stable Phase Change Materials for Thermal Energy Storage. *ACS Omega* **2023**, *8* (41), 38160–38169. https://doi.org/10.1021/acsomega.3c04311.

(13) Tselana, B. M.; Muniyasamy, S.; Ojijo, V. O.; Mhike, W. Melt Processible Biodegradable Blends of Polyethylene Glycol Plasticized Cellulose Diacetate with Polylactic Acid and Polybutylene Adipate-Co-Terephthalate. *J. Polym. Environ.* **2023**, *31* (11), 4891–4908. https://doi.org/10.1007/s10924-023-02925-8.

(14) Li, C.; Zhang, J.; Han, J.; Yao, B. A Numerical Solution to the Effects of Surface Roughness on Water–Coal Contact Angle. *Sci. Rep.* **2021**, *11* (1), 1–12. https://doi.org/10.1038/s41598-020-80729-9.

(15) Vivian, A.; Soumoy, L.; Fusaro, L.; Fiorilli, S.; Debecker, D. P.; Aprile, C. Surface-Functionalized Mesoporous Gallosilicate Catalysts for the Efficient and Sustainable Upgrading of Glycerol to Solketal. *Green Chem.* **2021**, *23* (1), 354–366. https://doi.org/10.1039/d0gc02562c.

(16) Gaballa, S. A.; Naguib, Y. W.; Mady, F. M.; Khaled, K. A. Polyethylene Glycol : Properties , Applications , and Challenges. **2024**, *7*, 26–36.

(17) Park, J. C.; Ito, T.; Kim, K. O.; Kim, K. W.; Kim, B. S.; Khil, M. S.; Kim, H. Y.; Kim, I. S. Electrospun Poly(Vinyl Alcohol) Nanofibers: Effects of Degree of Hydrolysis and Enhanced Water Stability. *Polym. J.* **2010**, *42* (3), 273–276. https://doi.org/10.1038/pj.2009.340.

(18) Rajasekharan, R.; Bahuleyan, A. K.; Madhavan, A.; Philip, E.; Sindhu, R.; Binod, P.; Kumar Awasthi, M.; Pandey, A. Neem Extract–Blended Nanocellulose Derived from Jackfruit Peel for Antibacterial Packagings. *Environ. Sci. Pollut. Res.* **2023**, *30* (4), 8977–8986. https://doi.org/10.1007/s11356-022-20382-z.

(19) Guo, Z.; Lin, Q. Coupling Reaction of CO2 and Propylene Oxide Catalyzed by DMC with Co-Complexing Agents Incorporated via Ball Milling. *J. Mol. Catal. A Chem.* **2014**, *390* (August 2014), 63–68. https://doi.org/10.1016/j.molcata.2014.03.006.

(20) Zhao, Y.; Xia, X.; Zhou, J.; Huang, Z.; Lei, F.; Tan, X.; Yu, D.; Zhu, Y.; Xu, H. Thermoresponsive Behavior of Non-Isocyanate Poly(Hydroxyl)Urethane for Biomedical Composite Materials. *Adv. Compos. Hybrid Mater.* **2022**, *5* (2), 843–852. https://doi.org/10.1007/s42114-021-00379-x.

(21) Morales Cepeda, A. B.; Macclesh del Pino, L. A.; Ramos Galvan, C. E.; González Pedraza, E. J.; Aguilera Vazquez, L. Synthesis of Glycerol Carbonate from Glycerin with CaCO3 in a Batch Reactor. *Des. Monomers Polym.* **2022**, *25* (1), 25–31. https://doi.org/10.1080/15685551.2022.2037215.

(22) Boccuzzi, F.; Chiorino, A.; Manzoli, M. FTIR Study of Methanol Decomposition on Gold Catalyst for Fuel Cells. *J. Power Sources* **2003**, *118* (1–2), 304–310. https://doi.org/10.1016/S0378-7753(03)00075-2.

(23) Mathew, A.; Kurmvanshi, S.; Mohanty, S.; Nayak, S. K. Sustainable Production of Polyurethane from Castor Oil, Functionalized with Epoxy- and Hydroxyl-Terminated Poly(Dimethyl Siloxane) for Biomedical Applications. *J. Mater. Sci.* **2018**, *53* (5), 3119–3130. https://doi.org/10.1007/s10853-017-1757-3.
